# Genome Wide SSR High Density Genetic Map Construction from an Interspecific Cross of *Gossypium hirsutum* × *Gossypium tomentosum*

**DOI:** 10.3389/fpls.2016.00436

**Published:** 2016-04-13

**Authors:** Muhammad K. R. Khan, Haodong Chen, Zhongli Zhou, Muhammad K. Ilyas, Xingxing Wang, Xiaoyan Cai, Chunying Wang, Fang Liu, Kunbo Wang

**Affiliations:** ^1^State Key Laboratory of Cotton Biology Institute of Cotton Research, Chinese Academy of Agricultural SciencesAnyang, China; ^2^Plant Breeding and Genetics Division, Nuclear Institute for Agriculture and BiologyFaisalabad, Pakistan; ^3^Cotton Sciences Research Institute of Hunan/National Hybrid Cotton Research Promotion CenterChangde, China; ^4^National Agricultural Research CentreIslamabad, Pakistan

**Keywords:** genetic map, interspecific cross, *Gossypium tomentosum*, wild cotton, SSR primer pairs

## Abstract

A high density genetic map was constructed using F_2_ population derived from an interspecific cross of *G. hirsutum* × *G. tomentosum*. The map consisted of 3093 marker loci distributed across all the 26 chromosomes and covered 4365.3 cM of cotton genome with an average inter-marker distance of 1.48 cM. The maximum length of chromosome was 218.38 cM and the minimum was 122.09 cM with an average length of 167.90 cM. A sub-genome covers more genetic distance (2189.01 cM) with an average inter loci distance of 1.53 cM than D sub-genome which covers a length of 2176.29 cM with an average distance of 1.43 cM. There were 716 distorted loci in the map accounting for 23.14% and most distorted loci were distributed on D sub-genome (25.06%), which were more than on A sub-genome (21.23%). In our map 49 segregation hotspots (SDR) were distributed across the genome with more on D sub-genome as compared to A genome. Two post-polyploidization reciprocal translocations of “A2/A3 and A4/A5” were suggested by seven pairs of duplicate loci. The map constructed through these studies is one of the three densest genetic maps in cotton however; this is the first dense genome wide SSR interspecific genetic map between *G. hirsutum* and *G. tomentosum*.

## Introduction

The cotton genus *Gossypium* consists of 50 species (Fryxell, 1992; Stewart, 1995; Ma et al., 2008), five are allotetraploid found in the New World with 26 pair of chromosomes (2n = 4x = 52; 13 A− and 13 D−) while 45 belongs to Old World with 13 pair of chromosomes (2n = 2x = 26); (Stewart, 1995; Brubaker et al., 1999a; Zhang et al., 2005). Evolution and diversity studies of genus *Gossypium* provide the basic knowledge of morphological diversity of the genus and plant biology which can help in the better utilization of genetic resources (Wendel et al., 2009).

The new advances in the molecular biology provide new approaches like genomics to construct molecular map of important traits that will divulge the genetic architecture of the traits and help in marker assisted selection (MAS) for speedy cotton improvement. The use of DNA markers in MAS can unleash the avenue toward robust crop improvement (Burr et al., 1983; Tanksley et al., 1988; Xu and Crouch, 2008) especially complex traits like fiber quality (Kohel et al., 2001) through the indirect selection of target traits.

More than 30 genetic maps have already been published in cotton; most of them are based on interspecific crosses of domesticated tetraploid species namely *G. hirsutum* and *G. barbadense* (S1 Table; Jiang et al., 1998; Zhang et al., 2002; Lacape et al., 2003, 2009; Nguyen et al., 2004; Rong et al., 2004; Guo et al., 2007; He et al., 2007). The interspecific tetraploid genetic maps are useful in understanding genome structure and exploring the genetics basis of important agronomic characters and also provide the basis for finding new DNA markers for further high density maps (Guo et al., 2007; Zhang et al., 2008; Yu et al., 2011).

In cotton, first detailed genetic linkage map, using RFLP molecular markers, was published by Reinisch et al. (1994). The map was constructed from interspecific cross of *G. hirsutum* and *G. barbadense* using 57 F_2_ populations. In total 705 loci in 41 linkage groups covered the 4675 cM of cotton genome map. However, 14 linkage groups were assigned to chromosome numbers using aneuploid lines. It was also estimated that genetic distance of 1 cM of cotton genome corresponding to ~400 kb genomic DNA. In 2004, Rong et al. further refined this map using a large number of RFLP markers along with some SSR markers, the number of loci increased to 2584 covering 4448 cM. However, the extensive use of RFLP markers was not become popular, because of their limitations.

In 2002, 58 DHs were used to construct the first PCR-based molecular marker map (Zhang et al., 2002). Later on, the strain “Vsg” (*G. barbadense*) used as semigamy line to produce 73 DHs and backcross populations (140) were developed from *G. hirsutum* and *G. barbadense*, these two populations were used to construct two genetic maps and they were compared (Song et al., 2005). Since DH lines were relatively small, and very difficult to develop, so backcross populations have played the key role in the construction of genetic maps. In 2004, Han et al. constructed the map comprising 624 loci using BC populations and later on in 2006 they further include more loci up to 907 in the map to enhance its density 907 (Han et al., 2006), in the year 2007 and 2008 Guo et al. enriched twice the density of the map from 1790 to 2247 cM. Zhao et al. (2012) further augmented the density of the Guo et al. (2008) map to 3414 covering 3668 cM of cotton genome, which is currently the world's largest and most dense genetic map of cotton. There are almost 10 different kinds of markers used for linkage, nine of them are molecular markers and the only one is morphological markers. Among molecular markers, SSRs are the largest mapped molecular markers and account for 2734 SSR loci, covering 80% of the entire marker used till to-date (Zhao et al., 2012).

Another genetic map included 2316 loci covering 4419 cM is the world's second SSR markers dense genetic map (Yu et al., 2011). In addition, Yu and other published a cotton genetic map in 2012 using the 186 RILs derived from *G. hirsutum* (TM-1) and *G. barbadense* (3–79) that is also very representative of 2072 marker loci including 1825 SSR loci, but they are the first who use SNP markers in cotton genomics. So far published major cotton interspecific genetic maps are shown in S1 Table. Almost all cotton genetic maps are based on the *G. hirsutum*; *G. barbadense* and hybrids of wild populations.

The occurrence of segregation distortion in plants is universal, a process in which genotypic frequencies are skewed from the expected violating Mendelian segregation ratios and these deviations can't be evaluated by simple genetic methods (Lu et al., 2002; Song et al., 2005; Li et al., 2010). Segregation distortion is widespread in intra and interspecific crosses (Causse et al., 1994; Ulloa et al., 2002; Rong et al., 2004; Lacape et al., 2009; Yu et al., 2011; Zhao et al., 2012), and is a driving force in the evolution of species (Taylor and Ingvarsson, 2003). Mangelsdorf and Jones (1926) reported for the first time the occurrence of segregation in maize, using morphological markers and afterward McCouch et al. (1988) and Pereira et al. (1994) reported segregation skewness in rice, sorghum, and tomato, respectively. Many factors like pollen tube competition, pollen killer genes, selective fertilization, abortion, chromosome translocation etc. are the major causes of segregation distortion (Luo and Xu, 2003; Taylor and Ingvarsson, 2003; Li et al., 2007; Zhu et al., 2007).

In this study, high density genetic map of cotton is developed from *G. hirsutum* × *G. tomentosum* which will serve as an indispensable genomic resource for fine positioning of important traits, genome organization and function, map-based gene cloning, comparative genomic analyses in cotton. The MAS studies unveil that upland cotton has narrow genetic base resulting into low rate of polymorphism among them (Wendel et al., 1992; Van Esbroeck et al., 1998; Gutierrez et al., 2002; Saha et al., 2003). For the efficient utilization of genetic resources from wild cotton molecular breeding approaches need to be established. This requires better understanding at genomic level which is the key feature. It is therefore a dire need to construct high-density genetic linkage map between upland cotton and *tomentosum*.

## Results

### Parental polymorphisms

Out of total primers used, 7411 (42.94%) were genomic SSR (gSSRs) and 9848 (57.06%) were EST-SSR (Table 1). In total 3091 SSRs were polymorphic between parents with an average of 17.91%, of which 20.64% (1530) were gSSRs and produced 1516 loci whereas 1561 were eSSR with polymorphism rate of 15.85% generating 1628 loci. BNL series has the highest polymorphism rate (41.69%) followed by MGHES with a polymorphism rate of 40.48% generating 169 and 36 loci, respectively. Among the eSSR primers MON_CER series has second highest polymorphism rate (27.27%) ensuing MGHES (40.48%). In case of gSSR primers the second highest polymorphism rate (32.32%) was of gNBRI. However, NAU generated the maximum number of loci (667) followed by HAU and Mon_CGR engendering 483 and 355 number of loci, respectively. Over all the polymorphism rate of genomic SSR primers was greater than EST-SSR primers in this study (Table 1).

**Table 1:** **Rate of Polymorphism of all SSR used in the research**.

**Primers**	**No. of primers**	**Polymorphic primers**	**Dominant Loci**	**co-dominant Loci**	**Polymorphic loci**	**Polymorphic rate (%)**	**Mapped Loci**
**EST-SSR (eSSR)**							
HAU	3382	441	110	373	483	13.04	472
MGHES	84	34	8	28	36	40.48	36
MON_CER	121	33	4	31	35	27.27	35
MON_SHIN	295	61	8	61	69	20.68	67
MUSS	554	75	33	50	83	13.54	83
NBRI	1970	288^*^	14	107	121	14.62	118
NAU	3250	609	91	576	667	18.74	652
STV	192	20	4	18	22	10.42	21
Subtotal	9848	1561	272	1244	1516	15.85	1484
**Genomic SSR (gSSR)**							
BNL	379	158	28	141	169	41.69	167
CIR	392	52	12	42	54	13.27	54
CM	53	13	1	14	15	24.53	15
DOW	100	5	0	5	5	5.00	5
DPL	200	55	2	57	59	27.50	59
Gh	700	186	15	190	205	26.57	202
ICRC	428	71^*^	5	30	35	16.59	34
JESPR	309	44	7	41	48	14.24	47
MON_C2	93	18	2	16	18	19.35	18
MON_CGR	1244	321	33	322	355	25.80	352
MON_COT	70	18	2	19	21	25.71	20
MON_DC	465	59	7	53	60	12.69	59
MON_DPL	649	167	37	150	187	25.73	187
MUSB	1316	103	40	73	113	7.83	107
NBRI	263	85	9	83	92	32.32	92
TMB	750	175	50	142	192	23.33	191
Subtotal	7411	1530	250	1378	1628	20.64	1609
G. Total	17,259	3091	522	2622	3144	17.91	3093

* *All the available primers were screened and polymorphism identified as above but only 121 and 35 SSR primers for NBRI and ICRC were used respectively due to time constraint*.

### Population genotyping

**Figure 1:**
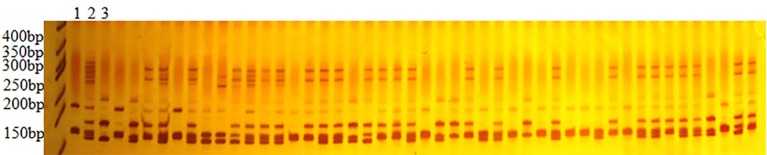
**Electrophoresis pattern of NAU2235 in F_**2**_ population 1–3 are CRI 12-2, F_**1**_ and ***G. tomentosum***, others are F_**2**_ individuals**.

In total 3091 polymorphic SSR primers were used to scrutinize for genotyping 188 F_2_ population that produced 3144 marker loci (Table 1). Figure 1 reveals the results of 8% polyacrylamide gel electrophoresis (PAGE) of NAU2235 primer, clearly showing that the primer produced the amplicons having two stable bands with different segregating pattern positions. These were denoted as NAU2235(a) and NAU2235(b) with higher and lower molecular weights, respectively. Of the 3144 marker loci, 522 loci accounting for 16.60% of the total were dominant while 83.40% (2622) were co-dominant (Table 1). From these 522 dominant loci 227 (43.45%) and 295 (56.51%) loci received alleles from CIR 12-2 and *G. tomentosum*, respectively. Of the total 1516 eSSR, 1224 loci (82.06%) were codominant while 272 (17.94%) were dominant loci. In case of the total 1628 gSSR, 1378 (84.64%) and 250 (15.36%) were co-dominant and dominant loci, respectively. It was observed that gSSR revealed more number of co-dominant loci than eSSR. Out of total 3144 polymorphic loci 48.22% were eSSR and 51.78% were gSSR.

### Genetic map features

In this study we used JoinMap 4.0 (Stam, 1993) mapping software for linkage analysis of 3144 polymorphic loci data (Table 2). The genetic linkage map comprises 3093 SSR marker loci that were mapped to the 26 chromosomes of cotton using 2823 SSR primers. The 51 loci did not make any linkage group and were not mapped because of missing data and highly skewed segregation. Of these markers 1354 were eSSR pairs wherein 1235 pairs amplified single loci, 111 pairs generated two loci each (222), five pair amplified three loci each (15), three pairs amplified quadruplicate loci each (12), all together the eSSR primers produced 1484 polymorphic loci that were mapped on the 26 chromosomes. The 1469 gSSR pairs of primers amplified 1609 marker loci mapped of which 1338 pairs were single locus, 123 pairs amplified two loci producing 246 marker loci, seven pairs amplified three loci (Guo et al., 2007), and only one amplified four loci. Out of 1484 eSSR loci, 691 were distributed in At genome, and 793 were distributed in Dt genome; while of the 1609 gSSR marker loci, 798 and 811 were located in At and Dt genomes, respectively (Table 2). Of the 3093 mapped loci, 2580 were co-dominant loci, with 1245 and 1335 distributed on At and Dt genomes, respectively, whereas 513 were dominant loci of which 225 loci received alleles from CRI 12-2 and 288 from *G. tomentosum*.

**Table 2:** **Main Characteristics of genetic map**.

**Chromosome**	**No. of marker loci**	**Recombination or map Size (cM)**	**Avg. distance b/w loci (cM)**	**Smallest gap (cM)**	**Largest gap (cM)**	**No. of gaps (>10 cM)**	**No. of skewed/SD loci**	**Percentage of skewness/SD %**	**No. of Dominant loci**	**No. of Co-dominant loci**	**eSSR**	**gSSR**
**AT SUB-GENOME**												
Chr.01(A01)	158	208.76	1.33	0.01	15.96	2	38	24.05	44	114	60	98
Chr.02(A02)	82	147.34	1.8	0.00	14.52	2	25	30.49	14	68	35	47
Chr.03(A03)	100	145.17	1.45	0.00	11.87	3	18	18	13	87	42	58
Chr.04(A04)	65	137.73	2.12	0.00	12.93	2	9	13.85	9	56	32	33
Chr.05(A05)	149	179.45	1.2	0.03	05.23	0	18	12.08	18	131	78	71
Chr.06(A06)	90	132.35	1.47	0.00	10.03	1	41	45.56	16	74	37	53
Chr.07(A07)	108	142.94	1.32	0.00	11.72	1	17	15.74	16	92	53	55
Chr.08(A08)	113	185.87	1.64	0.00	13.34	2	8	7.08	15	98	55	58
Chr.09(A09)	113	192.8	1.71	0.00	06.98	0	17	15.04	13	100	54	59
Chr.10(A10)	101	164.37	1.63	0.11	11.13	3	4	3.96	13	88	47	54
Chr.11(A11)	192	208.77	1.09	0.02	11.83	2	55	28.65	47	145	94	98
Chr.12(A12)	125	187.58	1.5	0.00	10.86	1	62	49.6	16	109	63	62
Chr.13(A13)	93	155.88	1.68	0.11	14.01	1	11	11.83	10	83	41	52
Subtotal	1489	2189.01	1.53	0.02	11.57	20	323	21.23	244	1245	691	798
**DT SUB-GENOME**												
Chr.15(D01)	129	172.32	1.31	0.00	10.57	1	36	27.91	31	98	68	61
Chr.14(D02)	122	205.21	1.68	0.00	14.07	3	19	15.57	14	108	69	53
Chr.17(D03)	81	122.09	1.51	0.00	13.22	1	16	19.75	24	57	37	44
Chr.22(D04)	68	167.68	2.47	0.00	16.30	2	23	33.82	11	57	32	36
Chr.19(D05)	215	218.38	1.02	0.00	05.28	0	43	20	47	168	118	97
Chr.25(D06)	124	153.99	1.32	0.00	10.60	1	59	47.58	23	101	53	71
Chr.16(D07)	121	161.03	1.33	0.00	09.09	0	12	9.92	14	107	67	54
Chr.24(D08)	139	150.48	1.08	0.00	05.56	0	26	18.71	25	114	71	68
Chr.23(D09)	113	160.95	1.42	0.00	07.49	0	24	21.24	13	100	55	58
Chr.20(D10)	125	149.09	1.19	0.13	08.61	0	35	28	21	104	52	73
Chr.21(D11)	147	209.59	1.43	0.00	13.07	1	41	27.89	20	127	67	80
Chr.26(D12)	121	169.66	1.4	0.02	08.41	0	23	19.01	12	109	54	67
Chr.18(D13)	99	135.82	1.37	0.09	09.63	0	36	36.36	14	85	50	49
Subtotal	1604	2176.29	1.43	0.02	10.15	9	393	25.06	269	1335	793	811
Grand total	3093	4365.30	1.48	0.02	10.86	29	716	23.14	513	2580	1484	1609

**Figure 2:**
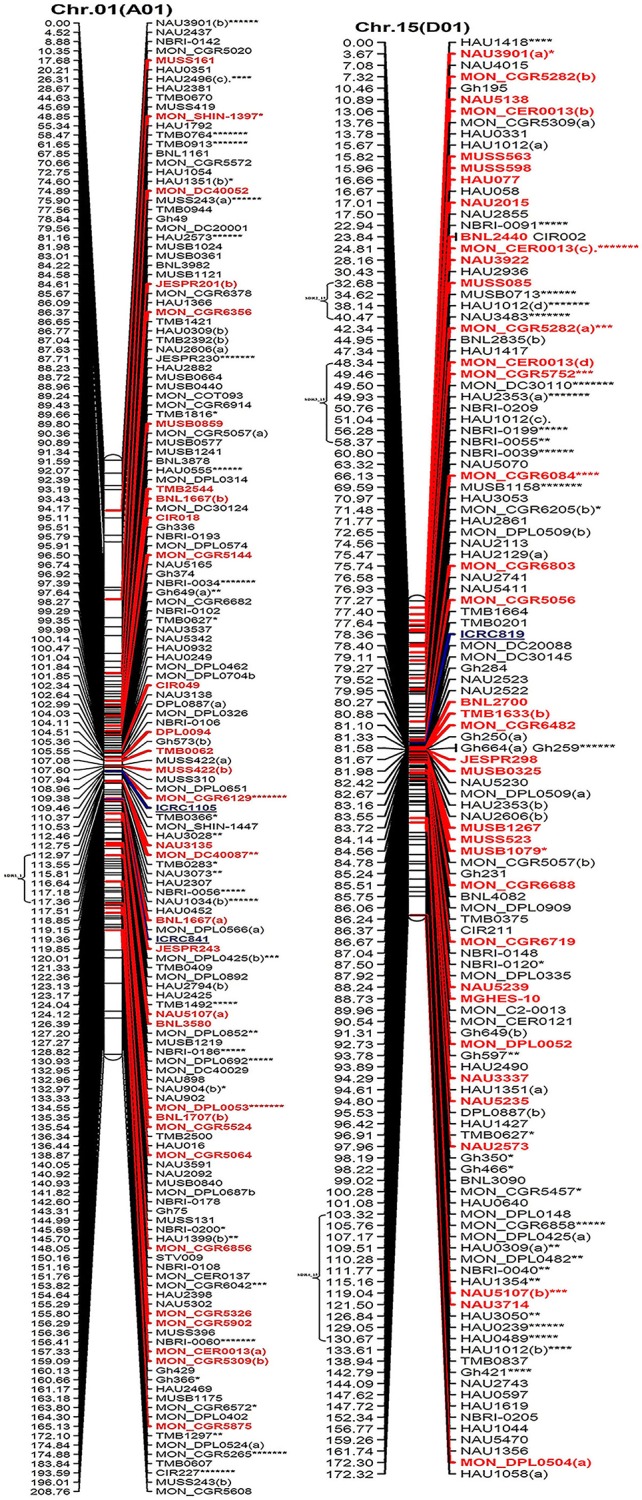

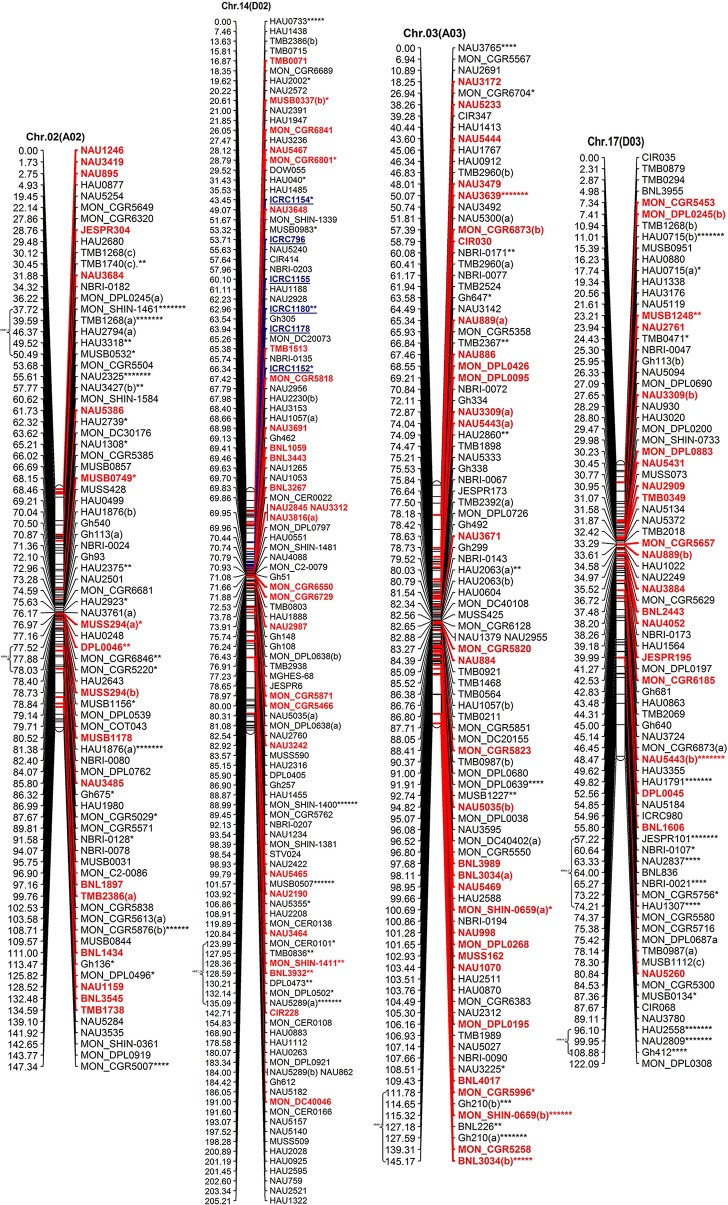

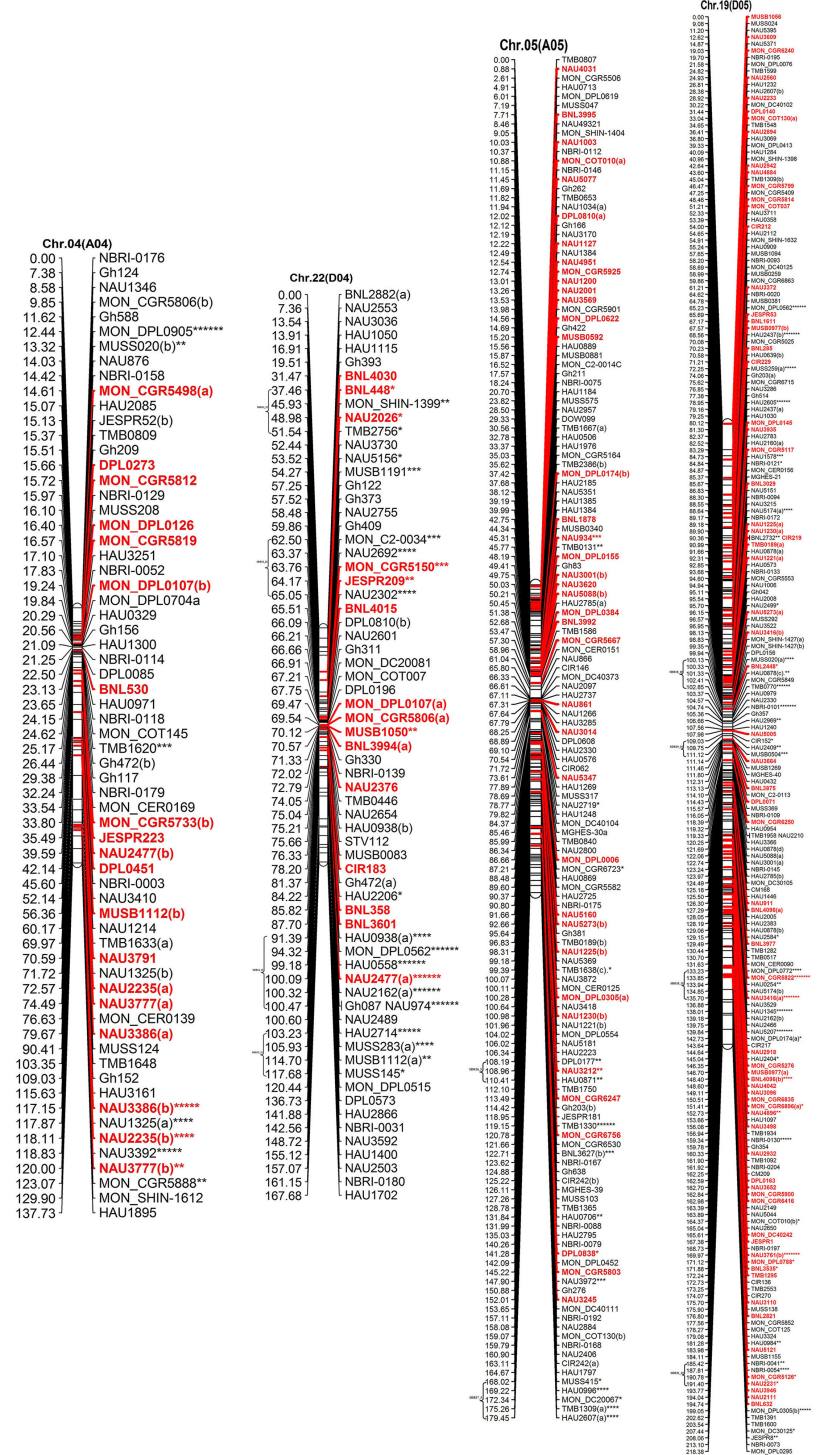

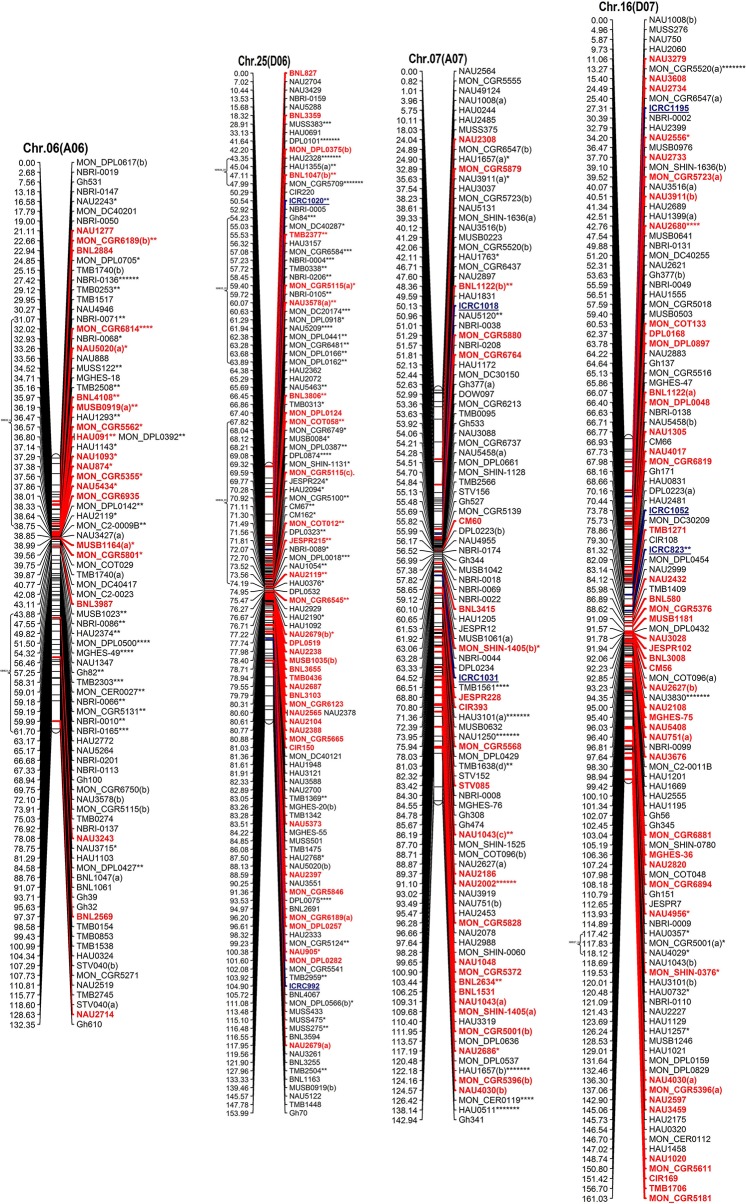

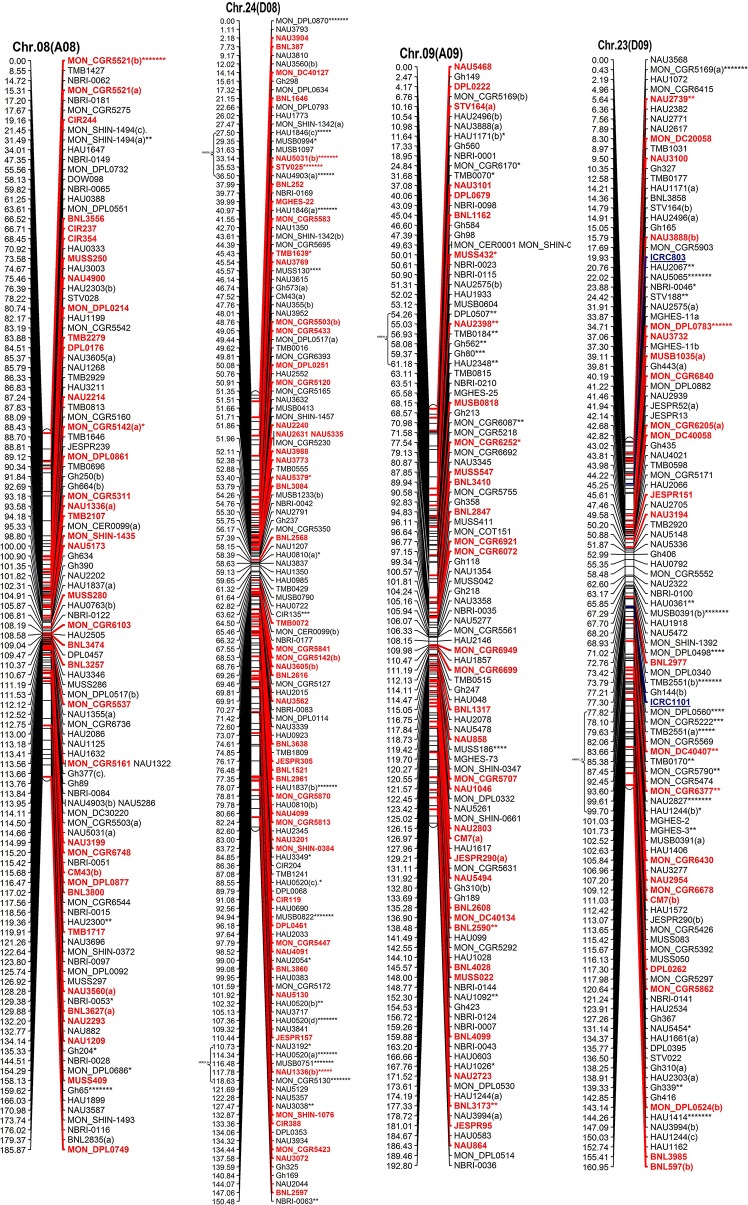

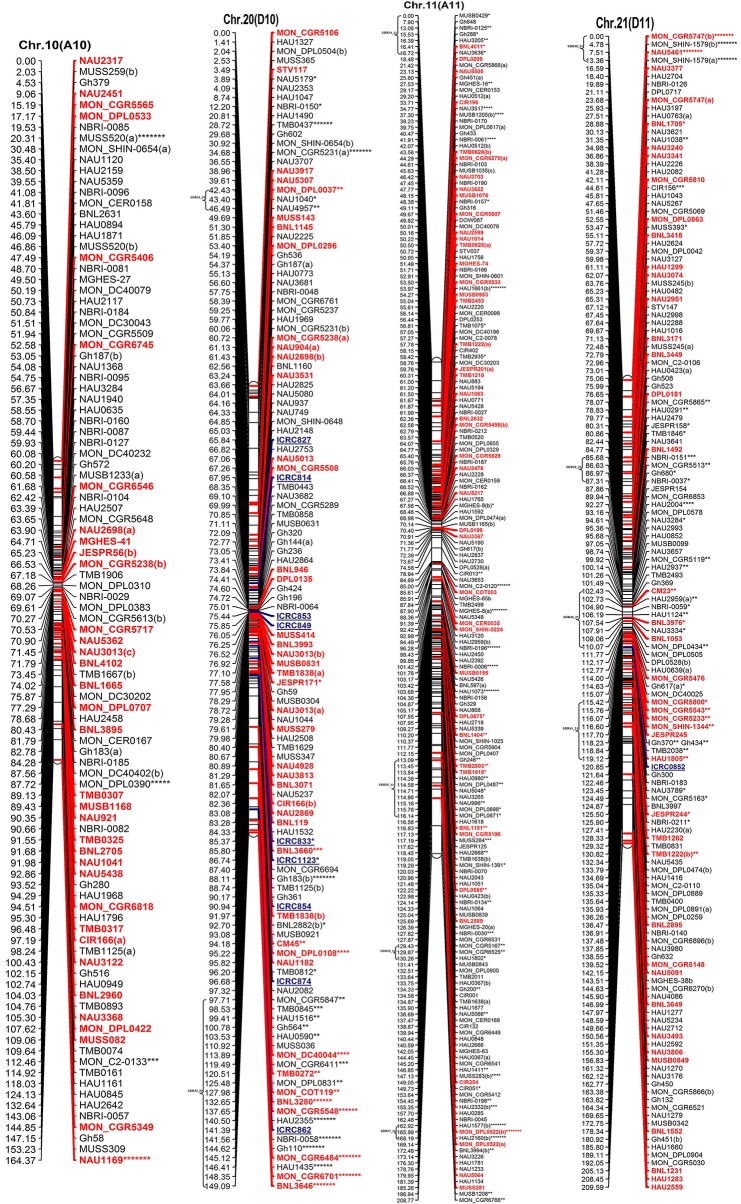

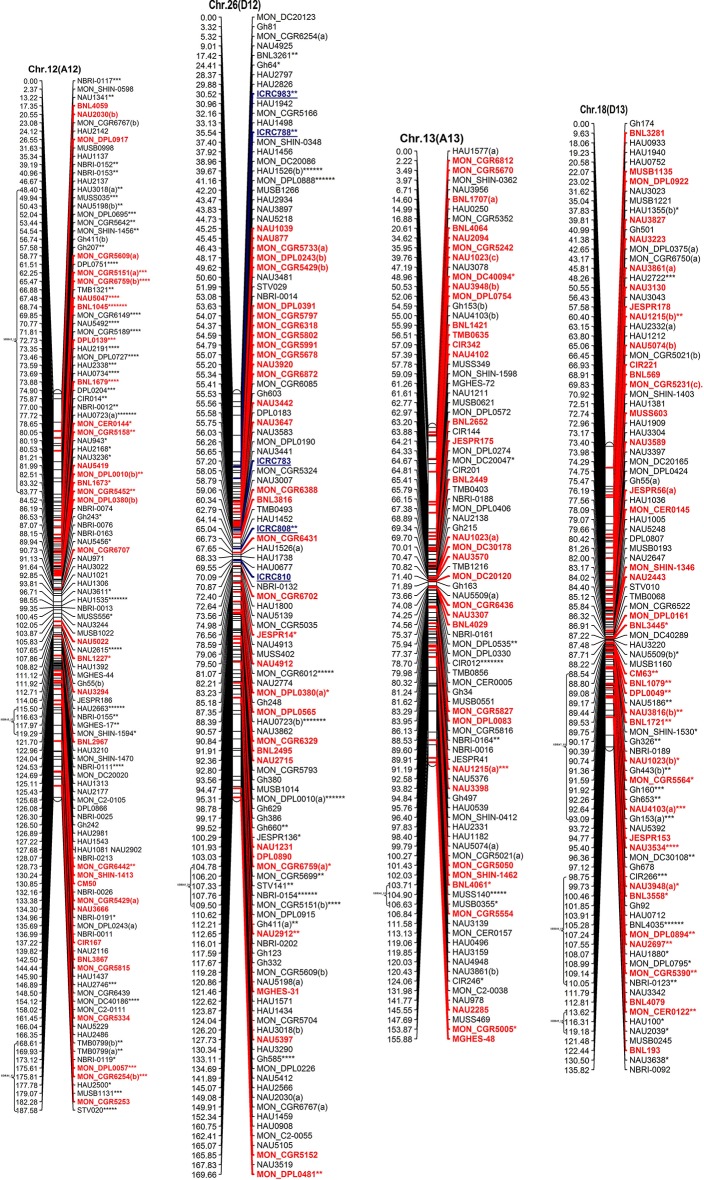
**The interspecific genetic map of the F_**2**_ population (CRI12-2 × ***G. tomentosum***)**. Genetic linkage map of allotetraploid cotton presented as 13 each At and Dt sub-genome homeologous chromosomes. The names of loci are shown on the right, and the positions of the loci are shown, in Kosambi centiMorgan (cM), on the left. The newly developed primer's name are underlined and blue in color whereas, markers showing segregation distortion are indicated by asterisks (^*^P < 0.05, ^**^P < 0.01, ^***^P < 0.005, ^****^P < 0.001, ^*****^P < 0.0005, ^******^P < 0.0001, *******P < 0.00005). The already anchored markers loci by other scientists are bold and red in color. For NBRI original names please see S2 Table.

All the mapped loci spanned 4365.30 cM with an average inter-marker distance of 1.48 cM (Table 2, Figure 2). The average length of chromosome in this map is 167.90 cM, containing an average number of 118.96 loci. Among At and Dt sub-genomes the chromosome Chr.19(D05) is the longest chromosome spanning 218.38 cM, with highest number of loci (215) on a chromosome; while shortest chromosome is Chr.17 (D03) covering a distance of 122.09 cM, containing 81 loci. On the other hand Chr.04 (A04) contains the minimum number of loci that is only 65 spanning a distance of 137.73 cM. The largest inter-marker distance is 2.47 cM on Chr.22 (D04) and the minimum is 1.02 cM on Chr.19 (D05) which is also the densest chromosome in terms of number of loci. The minimum marker interval is 0 cM and the biggest inter-loci gap is 16.3 cM on Chr.22 (D04); in total there are 29 gaps >10 cM with 20 and 9 gaps distributed on At and Dt sub-genomes, respectively.

The At sub-genome embraces 1489 loci covering a total genetic distance of 2189.01 cM with an average marker interval of 1.53 cM having largest and smallest interval of 2.12 and 1.09 cM, respectively. The longest chromosome, as far as recombination frequency is concerned, is Chr.11(A11) which covers 208.77 cM with 192 loci followed by Chr.01(A01) that spans 208.76 cM with 158 marker loci. There are 20 gaps >10 cM and largest gap of 15.96 cM is on Chr.01(A01) in the At sub-genome.

The Dt sub-genome comprises of 1604 loci which span a genetic distance of 2176.29 cM with an average marker interval of 1.43 cM having largest and smallest interval of 2.47 and 1.02 cM, respectively. The longest chromosome, as far as recombination frequency is concerned, is Chr.19 (D05) as mentioned before followed by Chr.21 (D11) that spans 209.59 cM with 147 marker loci. There are nine gaps >10 cM and largest gap in the Dt sub-genome is 16.3 cM on Chr.22(D04).

SSRs markers are not equally distributed among At and Dt sub-genomes with more gSSRs and eSSRs on the Dt sub-genome as inferred from above results. The more gSSRs are distributed on Chr.11(A11), Chr.19(D05), Chr.21(D11), Chr.25(D06), Chr.05(A05), and Chr.24(D08) while more eSSRs are distributed on Chr.19(D05), Chr.11(A11), Chr.05(A05), Chr.24(D08), Chr.14(D02), Chr.15(D01), and Chr.21(D11). The distribution of gSSRs and eSSRs on each chromosome is also differential. The gSSR and eSSR are almost equally distributed on Chr.18(D13), Chr.12(A12), Chr.04(A04), Chr.11(A11), Chr.24(D08), Chr.07(A07), and Chr.23(D09) with a difference of <3% however their distribution is not same on Chr.01(A01) and Chr.06(A06) with maximum differences of 24 and 18%, respectively (Figure 2).

### Characteristics of distorted segregation markers

**Figure 3:**
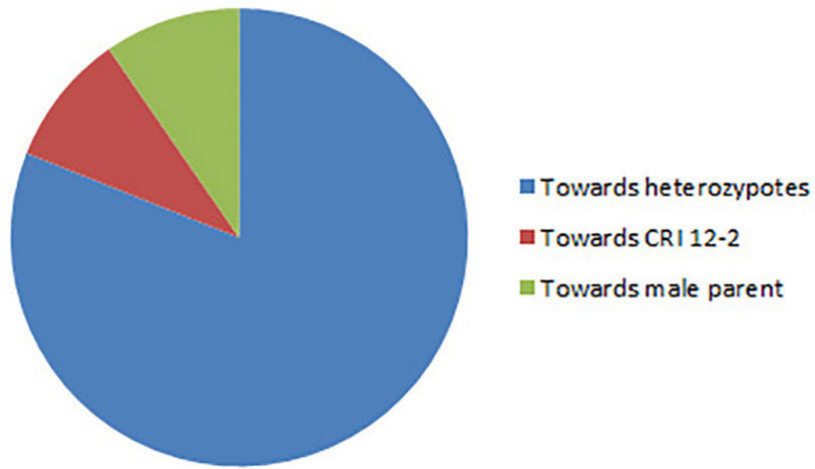
**Trend of distorted loci**.

Altogether 716 revealed skewness from normal Mendelian ratio called segregation distortion accounting for 23.14% distortion of the total mapped loci. Of the total 716 loci, 67 loci segregate toward the CIR 12-2 allele and 580 loci toward the heterozygous allele. According to the type of SSR i.e., 22.64% gSSR and 23.62% eSSR are distorted. These distorted loci are not evenly distributed on the 26 cotton chromosomes ranging from 4 to 62% on each chromosome. Dt sub-genome has more distorted loci, 393 or about 25.06%, than the At sub-genome which consisted of 323 (21.23%) distorted loci. Most of the segregation distortion loci are clustered on specific chromosomal segments i.e., segregation distortion region, SDR, as referred by Yu et al. (2011) and Zhao et al. (2012). A total of 49 SDRs were found of which six tend toward maternal parent CRI 12-2, two favor male parent *G. tomentosum*, and the remaining 41 segregate toward heterozygotes. Of the total SDRs 18 are on the At sub-genome and 31 on the Dt sub-genome. The chromosomes with the most distorted loci were Chr.11(A11), Chr.22(D04), Chr.19(D05), and Chr.21(D11). The distorted loci showed a phenomenon in which loci skewing toward the same allele appeared on the same chromosomes or within the same SDRs e.g., Chr.02(A02), Chr.03(A03), Chr.13(A13), Chr.14(D02), Chr.17(D03), and Chr.18(D13; Figure 2). SDR22_6, SDR25_26, SDR26_25, SDR33_20, SDR42_12, and SDR47_18 are the biggest SDRs and they all showed distortion toward the heterozygote (Figure 3). In this study, ~40% of SDRs are concentrated toward the end of the linkage group while ~30% of the SDR are located in the centromeric region. The smallest proportion of distorted loci is on Chr.10(A10) with 3.96%, and the largest proportion i.e., 49.6% on Chr.12(A12). But it is interesting that chromosomes; Chr.12(A12), Chr.25(D06), and Chr.06(A06) have the distortion ratios 49.6, 47.58, and 45.56%, respectively, which is far greater than distortion ratio on other chromosomes and the entire genome has higher segregation ratio (23.14%) than other maps (Table 2).

### Duplication, rearrangement, and translocation

In our map, 250 SSR primer pairs amplified two or more loci and collectively produced 520 multiple loci, with 468 duplicated, 36 triplicated, and 16 quadruplicated loci (Table 3 and S3 Table). Of these, 238 (50.85%) duplicated loci bridged the 13 homeologous At/Dt chromosomes. The remaining 230 duplicated loci were present between non-homeologous chromosomes and also have intra-chromosomal relationship i.e., within the same sub-genome. Of the 230 loci 44.34% loci were located on the same chromosome while 55.65% loci were present on different chromosomes (non-homeologous). This result revealed that during the evolutionary process multiple rounds of duplication both intra-chromosomal and inter-chromosomal occurred (Zhao et al., 2012). An intra-chromosomal duplications observed in chromosome 11 (A11), where five markers viz. HAU0367, HAU0512, MGHES-8, MGHES-8, MON_DPL0522, and TMB0628 each divulged duplicate loci and their recombination rates on an average are 10 cM.

**Table 3:** **Summary of multiple loci anchored on 26 homeologous and non-homeologous chromosomes**.

**Relationship**		**Chromosome**	**Duplicate loci**
Two loci	Homeologous chromosomes	Chr.01–Chr.15	20
		Chr.02–Chr.14	02
		Chr.03–Chr.17	10
		Chr.04–Chr.22	08
		Chr.05–Chr.19	30
		Chr.06–Chr.25	10
		Chr.07–Chr.16	34
		Chr.08–Chr.24	26
		Chr.09–Chr.23	18
		Chr.10–Chr.20	16
		Chr.11–Chr.21	18
		Chr.12–Chr.26	28
		Chr.13–Chr.18	18
	Subtotal		238
	Intra-chromosomal		102
	Non-homeologous		128
Three loci	Homeologous- Intra-chromosomal	–	15
	Homeologous- Non-homeologous		9
	Intra-chromosomal- Non-homeologous		6
	Intra-chromosomal		3
	Non-homeologous		3
	Subtotal		36
Four loci	Homeologous- Intra-chromosomal		4
	Intra-chromosomal- Non-homeologous		4
	Intra-chromosomal		8
	Subtotal		16
	Grand Total		520

Moreover, two post-polyploidization reciprocal translocations of A2/A3 and A4/A5 were suggested by seven pairs of duplicate loci in the At sub-genome with two pairs of duplicated loci were identified between A3 and D2 chromosomes, three pairs on A2 and D3, one pair of duplicate loci on each A5/D4 and A4/D5. The marker HAU2794 produced duplicate loci between Chr.01(A01) and Chr.02(A02), from which we can deduce a probable dividing line for the reciprocal translocation in these two At sub-genome chromosomes. However, it needs more dense mapping data in the vicinity of HAU2794 (S1 Table).

## Discussion

### Choice of parental material

It will be easier for us to dig up enough quantity of polymorphic primers if parents differ for one or more traits of interest. Secondly, high purity in parental materials is required to avoid impure residual heterozygosity which would lead to confusing population genotyping, resulting into difficult to determine linkage groups (Cloutier et al., 1995). Thirdly, hybrid offspring fertility must be considered, because higher rate of infertility not only hinder the production of appropriate segregating populations like F_2_ but may increase rate of segregation distortion.

In this study Upland cotton CRI 12-2 was used as female parent and one of the tetraploid wild cotton namely *G. tomentosum* as male parent. We selected the CRI 12-2 due to its verticillium disease resistance, high yield with superior fiber quality, wider adaptability and medium early maturing habit.

The main reason to select ***G. tomentosum*** wild cotton is to utilize the desirable exotic genes for the improvement of upland cotton and to enrich the germplasm. The MAS studies unveil that upland cotton has narrow genetic base resulting into low rate of polymorphism among them (Wendel et al., 1992; Van Esbroeck et al., 1998; Gutierrez et al., 2002; Saha et al., 2003). It has many unique agronomic traits that need to be introgressed into the upland cotton for developing superior cultivars.

For the efficient utilization of genetic resources from wild cotton molecular breeding approaches need to be established. This requires better understanding at genomic level and for economical utilization of desirable genes from *tomentosum*. It is therefore a dire need to construct high-density genetic linkage map between upland cotton and *tomentosum*.

### Segregation distortion

Highly dense maps offer the means to look across the whole genome for skewed loci (Causse et al., 1994; Harushima et al., 1996). However; it impinges on the development of genetic map and QTL detection (Zhu et al., 2007). In this study, out of 3093 mapped loci, 716 loci revealed the phenomenon of segregation distortion accounting for 23.14% which is significantly higher than Yu et al. (2011) and Zhao et al. (2012). One reason may be because these researchers used upland cotton and *barbadense* while we used *tomentosum* as second parent which is genetically more apart from upland resulting into more translocations, chromosomal rearrangements and other genomic structure variations leading to high segregation distortion. Studies have shown that wider genetic relationship would lead to increasing trends of segregation distortion (Kianian and Quiros, 1992). The other reason may be that different types of populations were used in these studies. In the present study F_2_ population was used, which has more segregation ratio and produce more classes of segregates than BC_1_, while they used BC_1_population. This study revealed that distorted markers are distributed on almost each chromosome, but are unevenly distributed in the various regions of chromosomes. Of the 716 skewed loci 323 mapped in At sub-genome and 393 in Dt sub-genome indicating that Dt subgenome has more segregation distortion than At subgenome. The reason may be that *Gossypium raimondii* is the putative contributor of the Dt sub-genome to *Gossypium hirsutum*. Wang et al. (2012) found that approximately 40% of the paralogous genes were present in more than one block, which suggests that this genome has undergone substantial chromosome rearrangement during its evolution. Segregation distortion is potential signatures of introgression segments in *G. hirsutum* mapping population. Therefore, these chromosomal aberrations may be one of the possible reasons for more distortion in Dt sub-genome than At sub-genome. Nucleo-cytoplasmic interactions may be the other reason as mention by Jiang C. X. et al. (2000). Other maps have also proved that Dt sub-genome showed more distortion (Yu et al., 2011). Most of the distorted loci skewed toward heterozygous alleles which are in concurrence with Zhao et al. (2012). Of all the distorted loci, 52.5**%** of the loci are located within the SDR. A total of 49 SDR are located across the genome with more on D sub-genome, and within one SDR all the distorted loci segregate in the same direction as reported by Yu et al. (2011). All large SDRs showed distortion toward the heterozygous allele as pointed out by Zhao et al. (2012). These SDRs provide the evidence for the presence of genetic loci which may be one of the causes of such distorted loci. These loci express at different times to trigger gametophyte and zygotic selection. In rice gametocidal gene has now been identified and mapped on the genome. In cotton the presence of such gametophyte gene need to be verified.

### High density genetic map

High density genetic maps are not only to unveil the genome structure and origin of evolution but are also gaining importance in the applied genetic and genomic research. In particular gene rich high density genetic maps would open horizon for genome sequencing, tagging agronomically important genes, QTL mapping, map based cloning and MAS. Low polymorphic rate within the upland cottons would restrain the development of high density cotton genetic map as the genome required huge number of marker to cover it fully (Park et al., 2005; Han et al., 2006; Guo et al., 2007; He et al., 2007). In lieu of this fact new gSSRs from *G. raimondii* were developed to cover more genome and construct high density map. In this study 51 loci could not be placed on the map because of missing data or presence of stutter bands due to DNA slippage during PCR complicating the interpretation of bands. Due to stumble bands heterozygotes perplexed with homozygotes leading to the cynicism in the estimate of heterozygosity. However, this can be subdued by adding genotypes of known band size. The high density cotton map was assembled based on the 3093 SSR mapped markers covering 4365.30 cM with an average inter-marker distance of 1.48 cM. In this study gSSR revealed more polymorphism than eSSR like many other studies including those of Han et al. (2006), Nguyen et al. (2004), and Reddy et al. (2001). Compared to already constructed dense genetic maps of Rong et al. (2004) including 2584 mapped markers loci at 1.72 cM interval covering 4447.9 cM; Guo et al. (2008) comprised of 2247 loci covering 3440.4 cM with an average inter loci distance of 1.58 cM; Yu et al. (2011) consisting of 2316 marker loci at 1.91 cM interval with map length of 4418.9 cM; Yu et al. (2012) having 2072 marker loci covering 3380 cM at an average distance of 1.63 cM between markers and Zhao et al. (2012) including 3414 mapped loci spanning 3667.62 cM with average inter loci distance of 1.08 cM., our map ranked second after Zhao et al. (2012) as far as total mapped loci are concerned but stands first in terms of mapped SSR loci (3093). In the present map Chr.19(D05) has the most loci while Chr.04(A04) contains the least marker hence the distribution of markers on chromosomes is similar to Yu et al. (2011) and Yu et al. (2012). More marker loci were distributed on the D sub-genome than on the A sub-genome in this map which is in conformity with the results of Guo et al. (2007); Yu et al. (2011), and Yu et al. (2012), while Rong et al. (2004) reported conversely. The A sub-genome covers longer distance (2189.01 cM) than the D sub-genome (2176.29 cM) in this study and the previous reports of Rong et al. (2004); Yu et al. (2011) and Yu et al. (2012) but Guo et al. (2007) revealed shorter A sub-genome. In the present high density map the average inter-marker distance of D sub-genome (1.43 cM) is lesser than A sub-genome (1.53 cM) which is in concurrence with the results of all three maps mentioned above. The number of gaps >10 cM in A sub-genome is higher than D sub-genome in this map like Yu et al., [28], but its reverse in case of Yu et al. (2011). Both gSSRs and eSSRs are distributed more in D sub-genome than A sub-genome, and Chr.19(D05) and Chr.11(A11) are the highest gSSR and eSSR containing chromosomes which is in agreement with Yu et al. (2011). Moreover, their distribution across the 26 chromosomes is also varied. This phenomenon of uneven dissemination of SSRs on both the sub-genomes and chromosomes may offer to ascertain SSR repeat motif distribution in the genome and eSSR sequences could be advantageous in the mapped based cloning and MAS. The SSRs designed in the present study from *G. raimondii* (D genome cotton) mostly (88.24%) mapped on D sub-genome which may help to study the evolution of tetraploid cotton e.g., 50% of the newly *raimondii-*derived SSR distributed on Chr.20(D10), Chr.14(D02) which indicates that these chromosomes may endure rigorous evolution.

Zhao et al. (2012) increased the Guo et al. (2008) map from 2247 to 3414 marker loci with enhancement of 1167 loci, which include 541 new loci on the A sub-genome and 626 new loci on the D sub-genome, however they increased map length of only 127.2 cM, and Rong et al. (2004) enhanced the marker loci in the Reinisch et al. (1994) map from 705 to 2584 but the covered map length decreased from 4675 to 4447.9 cM.

Mostly high density cotton genetic maps have been developed from interspecific crosses of upland and *barbadense* but in the present study wild cotton *G. tomentosum* was used in order to understand the genome structure, organization and evolution which will offer the basis of genome comparison leading to enrich the presently available cotton germplasm by incorporating superior genes especially disease resistance from wild genetic resources.

## Materials and methods

There should be no specific permissions were required for conducting experiments at National Wild Cotton long-term *in vivo* nursery, Sanya because this nursery was especially established by Institute of Cotton Research, Chinese Academy of Agricultural Sciences, China as experimental field for research purposes on wild cotton. It is further confirm that the field studies did not involve endangered or protected species.

### Plant materials

F_1_ hybrid was generated from an interspecific cross of *G. hirsutum* L var. CRI 12-2 (as female parent) and *G. tomentosum*, P0601211 (as male parent). By selfing of F_1_, 2022 F_2_ individuals were generated and sown in the field at National Wild Cotton long-term *in vivo* nursery, Sanya, China. From these 2022 F_2_ individuals only 188 immortalized plants were randomly selected to construct F_2_ population for the development of high density genetic map. In order to keep these selected plants as immortalized every autumn aboveground plant parts were cut off. Genomic DNA of parents, F_1_ and F_2_ population of 188 individuals was extracted from young leaf tissue, by CTAB DNA extraction procedure, as described by Zhang and Stewart (2000) with some modifications. PCR amplification was done on TAKARA Bio Inc. TP 600 thermal cycler and silver staining following the method described by Zhang et al. (2002).

### PCR primers

In total 17,259 pairs of SSR primers were analyzed to identify polymorphic markers between CRI 12-2 and *Gossypium tomentosum* (P0601211). Whereas, ICRC series of primers were independently designed primers in our laboratory using *G. raimondii* genome sequences obtained from scaffolds (data not presented here), the all other SSR primers form Cotton Marker Database (CMD; http://www.cottonmarker.org/) published on the primer (Table 4).

**Table 4:** **Description of all SSR primers**.

**Sr. Number**	**Primer**	**Number of gSSRs**	**Number of eSSRs**
1	BNL	379	–
2	CIR	392	–
3	CM	53	–
4	DPL	200	–
5	Gh	700	–
6	HAU	0	3382
7	JESPR	309	–
8	MGHES	0	84
9	Mon_C2	93	–
10	Mon_DPL	649	–
11	Mon_CGR	1244	–
12	Mon_CER	–	121
13	Mon_COT	70	–
14	Mon_SHIN	–	295
15	Mon_DC	465	–
16	MUSB	1316	–
17	MUSS	–	554
18	NAU	–	3250
19	STV	0	192
20	TMB	750	0
21	DOW	100	
22	NBRI	263	1970
23	ICRC	428	
	Subtotal	7411	9848
	Grand total	17,259	

### Marker data acquisition/genotyping

SSR data collections were performed manually for gel-based assays. Polymorphic markers were used to survey F_2_ mapping population. According to parents and F_1_-based authentication primers were identified as dominant or co-dominant. All co-dominant markers were scored using the Mendelian segregation ratio 1:2:1 and all the dominant markers were scored using the 3:1 Mendelian segregation ratio. SSR amplicons were encoded as “10” (only one upper band), “11” (two bands), and “01” (only one lower band) and vice versa. For dominant loci, “1” was scored for presence, and “0” for absence. In both cases, “−” was recorded as missing data which included the blurred or vague bands. If a marker formed multiple bands within the same gel but with different molecular weight, it means marker is segregating for multiple loci that marker is known as multi-allelic marker. Besides the main bands if the markers produced other stable bands with different segregation pattern from the main bands (multi-allelic marker), they were named separately by primer name followed by the letters (a), (b), (c), (d) as a suffix for differentiating between them.

### Map construction

JoinMap 4.0 was used in this study. Kosambi mapping function was used to convert recombination frequencies into map distances (centimorgan, cM). The maximum recombination rate is set to 0.40. LOD ≥ 3.0 is generally believed that there is a linkage between the two loci. We used the LOD ≥ 10 in order to classify all the 78 small linkage groups into 26 large linkage groups corresponding to all the 26 chromosomes. During this process some primers failed to get on linkage group. Mapchart 2.2 software was used to draw map using the map distances and loci obtained from JoinMap. Segregation distortion is a ubiquitous phenomenon in interspecific crosses, which distort the segregation ratio. χ^2^-test was used to determine the skewness in the segregation ratios.

### Chromosome assignments and nomenclature

Chromosome assignment was established by the common markers that were already anchored by other authors in previous publications according to CMD website (http://www.cottonmarker.org/cmap/index.shtml). Chromosomal nomenclature was used as mentioned by Guo et al. (2008) Nanjing Agricultural University i.e., SSR loci anchored on chromosomes (Chr.) 1–13 were designated to the A sub-genome (At), whereas loci confined to Chr. 14–26 were designated to the D sub-genome (Dt).

## Conclusion

The map constructed through these studies is one of the three densest genetic maps in cotton however; this is the first dense genome wide SSR interspecific genetic map between *G. hirsutum* and *G. tomentosum*. This map will play an important role in understanding the genome structure of *G. tomentosum* and also open the doors for further in-depth genome research such as fine mapping, map-based cloning, evolutionary studies, tagging genes of interest from wild relatives, MAS and comparative mapping not only in cotton but also with other species as well.

## Author contributions

KW, MK, and FL designed the experiments. MK and HC conceived the experiments and analyzed the results. MK carried out most of the experiments, MK and HC carried out all computational analyses. ZZ, MI, XW, XC, CW, FL participated in part of mapping experiments directly or indirectly like contributed reagents/materials/analysis tools etc. MK, HC, and MI drafted the manuscript and KW revised the manuscript. All authors read and approved the final manuscript.

### Conflict of interest statement

The authors declare that the research was conducted in the absence of any commercial or financial relationships that could be construed as a potential conflict of interest.

## Abbreviations

BC: Back Cross
cM: Centimorgan
DH: Doubled Haploid
DNA: Deoxyribonucleic Acid
eSSR: EST-SSR
gSSRs: Genomic SSR
EST: Expressed Sequence Tag
EST-SSRs: Expressed Sequence Tag derived-SSRs
F_1_: First Filial Generation
F_2_: Second Filial Generation
LOD: Likelihood Odds Ratio
CMD: Cotton Marker Database
MAS: Marker Assisted Selection
SDR: Segregation distortion region
PCR: Polymerase Chain Reaction
QTL: Quantitative trait loci
RFLP: Restriction Fragment Length Polymorphism
SSR: Simple Sequence Repeat
RIL: Recombinant Inbred Lines
SDR: Segregation Distortion Region
SNP: Single Nucleotide Polymorphism
spp.: Species.
