# Supplementary material for: Genome Wide SSR High Density Genetic Map Construction from an Interspecific Cross of Gossypium hirsutum × Gossypium tomentosum
Source: Front Plant Sci. 2016 Apr 13;7:436. doi: 10.3389/fpls.2016.00436 (PMC4829609; doi:10.3389/fpls.2016.00436)
Supplement: Supplementary file 1 [file Table1.DOCX]

**S1 Table Published genetic linkage maps of cotton using interspecific population**

| Species | Female Parent | Male Parent | Pop. Type | Pop. Size | Marker Type | No. of Loci | Genome coverage (cM) | Reference |
| --- | --- | --- | --- | --- | --- | --- | --- | --- |
| Gh/Gb | Palmeri | K-101 | F2 | 57 | RFLP | 705 | 4675 | Reinisch et al., 1994 |
| Gh/Gb | CAMD-E | Sea Island Seaberry | F2 | 271 | RFLP | 261 | 3767 | Jiang et al., 1998 |
| Gher/Ga | A2(=A1)97 | A247 | F2 | 58 | Isozyme, RFLP | 161 | 856 | Brubaker et al., 1999b |
| Gtri/Grai | *G.trilobum* | *G.raimondii* | F2 | 62 | RFLP | 306 | 1486 | Brubaker et al., 1999b |
| Gh/Gb | *G.hirsutum* mutants | Sea Island Seaberry | F2 | 180 | RFLP | 261 | 3664 | Jiang C. et al., 2000 |
| Gh/Gb | TM-1 | 3-79 | F2 | 171 | RFLP,RAPD | 355 | 4766 | Kohel et al., 2001 |
| Gh/Gb | Siv’on | F-177 | F2:3 | 214 | RFLP | 253 | 4675 | Saranga et al., 2001 |
| Gh/Gb | TM-1 | Hai7124 | DH | 58 | SSR,RAPD | 489 | 3315 | Zhang et al., 2002a |
| Gh/Gb | Guazuncho2 | VH8-4602 | BC1 | 75 | AFLP,SSR,RFLP,morpho- | 888 | 4400 | Lacape et al., 2003 |
| Gh/Gb | Guazuncho2 | VH8-4602 | BC1 | 75 | AFLP,SSR,RFLP, morpho- | 1160 | 5519 | Nguyen et al., 2004 |
| Gh/Gb | Acala-44 | Pima S-7 | F2 | 120 | AFLP,SSR,RFLP | 392 | 3287 | Mei et al., 2004 |
| Gh/Gb | Palmeri | K-101 | F2 | 57 | SSR,RFLP | 2584 | 4448 | Rong et al., 2004 |
| Gh/Gb | TM-1 | Hai7124 | BC1 | 140 | SSR, morpho- | 624 | 5644 | Han et al., 2004 |
| Gh/Gb | TM-1 | Hai7124 | BC1 | 140 | SSR | 442 | 4331 | Song et al., 2005 |
| Gb/GhGb | 'TM-1 | Hai7124 | DH | 73 | SSR | 444 | 3263 |  |
| Gh/Gb | TM-1 | 3-79 | RIL | 183 | SSR,CSR | 193 | 1277 | Park et al., 2005 |
| Gh/Gt | TMS-22 | WT936 | F2 | 82 | RFLP | 589 | 4979 | Waghmare et al., 2005 |
| Gh/Gb | Handan208 | Pima90 | F2 | 69 | SSRs,RAPDs, SRAPs | 566 | 5142 | Lin et al., 2005 |
| Gh/Gb | Guazuncho2 | VH8-4602 | BC1 | 75 | SSR, AFLP | 1160 | 5520 | Lacape et al., 2005 |
| Gh/Gb | TM-1 | Hai7124 | BC1 | 140 | SSR, morpho- | 907 | 5060 | Han et al., 2006 |
| Gh/Gb | TM-1 | 3-79 | RILs | 183 | SSR | 433 | 2126 | Frelichowski et al., 2006 |
| Gh/Gb | Handan208 | Pima 90 | F2 | 69 | SSR,RAPD,SRAP,REMAP | 1029 | 5472 | He et al., 2007 |
| Gh/Gb | TM-1 | Hai7124 | BC1 | 138 | SSR, SRAP | 1790 | 3426 | Guo et al., 2007 |
| Gh/Gb | TM-1 | Hai7124 | BC1 | 138 | SSR, SRAP | 2247 | 3540 | Guo et al., 2008 |
| Gh/Gb | Emian22 | Pima 3-79 | BC1 | 141 | SSR | 917 | 5452 | Zhang et al., 2008 |
| Gh/Gb | Emian22 | Pima 3-79 | BC1 | 141 | SSR | 2316 | 4419 | Yu et al., 2011 |
| Gh/Gb | TM-1 | 3-79 | RIL | 186 | SSR,SNP | 2072 | 3380 | Yu et al., 2012 |
| Gh/Gb | TM-1 | Hai7124 | BC1 | 138 | SSR, SRAP, REMAP, RT, AFLP,SNP,CAPS | 3414 | 3668 | Zhao et al, 2012 |

Gh: *G. hirsutum*, Gb: *G. barbadense*, Gher: *G. herbaceum*, Ga: *G. arboreum*, Gtri: *G. trilobum*, Grai: *G. raimondii*
