# Supplementary material for: Genome Wide SSR High Density Genetic Map Construction from an Interspecific Cross of Gossypium hirsutum × Gossypium tomentosum
Source: Front Plant Sci. 2016 Apr 13;7:436. doi: 10.3389/fpls.2016.00436 (PMC4829609; doi:10.3389/fpls.2016.00436)
Supplement: Supplementary file 2 [file Table2.DOCX]

**S2 Table. Coding list of NBRI primers used**

| **Sr. No.** | **EST-SSR (eSSR)** | | **Sr. No.** | **Genomic SSR (gSSR)** | |
| --- | --- | --- | --- | --- | --- |
|  | **Original Name** | **Code** |  | **Original Name** | **Code** |
| 1 | NBRI_Gh_A_5113 | NBRI-0001 | 1 | NBRI_Gh_A007 | NBRI-0007 |
| 2 | NBRI_Gh_A_EYI1BW401A0ZW4 | NBRI-0002 | 2 | NBRI_Gh_A107 | NBRI-0008 |
| 3 | NBRI_Gh_A_EYI1BW401ARZB7 | NBRI-0003 | 3 | NBRI_Gh_B102 | NBRI-0025 |
| 4 | NBRI_Gh_A_EYI1BW401AULZ1-1 | NBRI-0004 | 4 | NBRI_Gh_B107 | NBRI-0026 |
| 5 | NBRI_Gh_A_EYI1BW401AULZ1-2 | NBRI-0005 | 5 | NBRI_Gh_B115 | NBRI-0027 |
| 6 | NBRI_Gh_A_EYI1BW401BRUJS-2 | NBRI-0006 | 6 | NBRI_Gh_C001 | NBRI-0035 |
| 7 | NBRI_Gh_A2_1113 | NBRI-0009 | 7 | NBRI_Gh_D_5346(a) | NBRI-0040 |
| 8 | NBRI_Gh_A2_1306 | NBRI-0010 | 8 | NBRI_Gh_D_5346(b) | NBRI-0041 |
| 9 | NBRI_Gh_A2_3431 | NBRI-0011 | 9 | NBRI_Gh_D001 | NBRI-0044 |
| 10 | NBRI_Gh_A2_3667 | NBRI-0012 | 10 | NBRI_Gh_E003-1(a) | NBRI-0057 |
| 11 | NBRI_Gh_A2_4904 | NBRI-0013 | 11 | NBRI_Gh_E003-1(b) | NBRI-0058 |
| 12 | NBRI_Gh_A2_878 | NBRI-0014 | 12 | NBRI_Gh_F029F | NBRI-0061 |
| 13 | NBRI_Gh_B_2021 | NBRI-0015 | 13 | NBRI_Gh_G035(a) | NBRI-0062 |
| 14 | NBRI_Gh_B_2834 | NBRI-0016 | 14 | NBRI_Gh_G035(b) | NBRI-0063 |
| 15 | NBRI_Gh_B_EYI1BW404HZC8N | NBRI-0018 | 15 | NBRI_Gh_H014 | NBRI-0064 |
| 16 | NBRI_Gh_B_EYI1BW404IBOTR | NBRI-0019 | 16 | NBRI_Gh_J021 | NBRI-0065 |
| 17 | NBRI_Gh_B_EYI1BW404IFHV5 | NBRI-0020 | 17 | NBRI_Gh_J040 | NBRI-0066 |
| 18 | NBRI_Gh_B_EYI1BW404IM5MZ | NBRI-0021 | 18 | NBRI_Gh_J045 | NBRI-0067 |
| 19 | NBRI_Gh_B_EYI1BW404IQ96G | NBRI-0022 | 19 | NBRI_Gh_K007 | NBRI-0068 |
| 20 | NBRI_Gh_B_EYI1BW404IS1WI | NBRI-0023 | 20 | NBRI_Gh_K011 | NBRI-0069 |
| 21 | NBRI_Gh_B_EYI1BW404JX74J | NBRI-0024 | 21 | NBRI_Gh_K036 | NBRI-0070 |
| 22 | NBRI_Gh_B2_659-2 | NBRI-0028 | 22 | NBRI_Gh_L010 | NBRI-0071 |
| 23 | NBRI_Gh_C_3005 | NBRI-0029 | 23 | NBRI_Gh_L015(a) | NBRI-0072 |
| 24 | NBRI_Gh_C_EYT27PB01A37GG | NBRI-0030 | 24 | NBRI_Gh_L021 | NBRI-0073 |
| 25 | NBRI_Gh_C_EYT27PB01AMGBS | NBRI-0031 | 25 | NBRI_Gh_L026 | NBRI-0074 |
| 26 | NBRI_Gh_C_EYT27PB01BWRQC | NBRI-0034 | 26 | NBRI_Gh_L029 | NBRI-0075 |
| 27 | NBRI_Gh_C2_14 | NBRI-0036 | 27 | NBRI_Gh_L035 | NBRI-0076 |
| 28 | NBRI_Gh_C2_1455 | NBRI-0037 | 28 | NBRI_Gh_L043 | NBRI-0077 |
| 29 | NBRI_Gh_C2_408 | NBRI-0038 | 29 | NBRI_Gh_PB_70-1 | NBRI-0078 |
| 30 | NBRI_Gh_D_4350 | NBRI-0039 | 30 | NBRI_Gh_PB_91 | NBRI-0079 |
| 31 | NBRI_Gh_D_EYT27PB02CZHSN | NBRI-0042 | 31 | NBRI_Gh_PC_81 | NBRI-0080 |
| 32 | NBRI_Gh_D_EYT27PB02D8CE6 | NBRI-0043 | 32 | NBRI_Gh_PD_21 | NBRI-0081 |
| 33 | NBRI_Gh_D2_2118 | NBRI-0045 | 33 | NBRI_Gh_A010 | NBRI-0097 |
| 34 | NBRI_Gh_D2_2168 | NBRI-0046 | 34 | NBRI_Gh_A105 | NBRI-0098 |
| 35 | NBRI_Gh_D2_2330 | NBRI-0047 | 35 | NBRI_Gh_A112 | NBRI-0099 |
| 36 | NBRI_Gh_E_1491 | NBRI-0048 | 36 | NBRI_Gh_B001 | NBRI-0107 |
| 37 | NBRI_Gh_E_2983 | NBRI-0049 | 37 | NBRI_Gh_B007(a) | NBRI-0108 |
| 38 | NBRI_Gh_E_EYT27PB03FLDF9 | NBRI-0050 | 38 | NBRI_Gh_C_EYT27PB01AX6Q0(a) | NBRI-0119 |
| 39 | NBRI_Gh_E_EYT27PB03FW1JT | NBRI-0051 | 39 | NBRI_Gh_C_EYT27PB01AX6Q0(b) | NBRI-0120 |
| 40 | NBRI_Gh_E_EYT27PB03G23YH | NBRI-0052 | 40 | NBRI_Gh_C111 | NBRI-0128 |
| 41 | NBRI_Gh_E_EYT27PB03GAY1V | NBRI-0053 | 41 | NBRI_Gh_C112 | NBRI-0129 |
| 42 | NBRI_Gh_E_EYT27PB03GDPRM | NBRI-0054 | 42 | NBRI_Gh_D_EYT27PB02EESBJ(b) | NBRI-0135 |
| 43 | NBRI_Gh_E_EYT27PB03HDVFT-1 | NBRI-0055 | 43 | NBRI_Gh_D002 | NBRI-0136 |
| 44 | NBRI_Gh_E_EYT27PB03HFOEI | NBRI-0056 | 44 | NBRI_Gh_D008 | NBRI-0137 |
| 45 | NBRI_Gh_E2_2226 | NBRI-0059 | 45 | NBRI_Gh_D010 | NBRI-0138 |
| 46 | NBRI_Gh_E2_763 | NBRI-0060 | 46 | NBRI_Gh_D111 | NBRI-0139 |
| 47 | NBRI_Gh_A_1546 | NBRI-0082 | 47 | NBRI_Gh_D112 | NBRI-0140 |
| 48 | NBRI_Gh_A_2329 | NBRI-0083 | 48 | NBRI_Gh_E005-1 | NBRI-0154 |
| 49 | NBRI_Gh_A_2721 | NBRI-0084 | 49 | NBRI_Gh_E006-1 | NBRI-0155 |
| 50 | NBRI_Gh_A_6098 | NBRI-0085 | 50 | NBRI_Gh_F002 | NBRI-0157 |
| 51 | NBRI_Gh_A_EYI1BW401AUW7X | NBRI-0086 | 51 | NBRI_Gh_F014b | NBRI-0158 |
| 52 | NBRI_Gh_A_EYI1BW401B1OR0 | NBRI-0087 | 52 | NBRI_Gh_F016 | NBRI-0159 |
| 53 | NBRI_Gh_A_EYI1BW401B6IW3 | NBRI-0088 | 53 | NBRI_Gh_F017 | NBRI-0160 |
| 54 | NBRI_Gh_A_EYI1BW401B6VIN | NBRI-0089 | 54 | NBRI_Gh_F020 | NBRI-0161 |
| 55 | NBRI_Gh_A_EYI1BW401BHE8E | NBRI-0090 | 55 | NBRI_Gh_F025 | NBRI-0162 |
| 56 | NBRI_Gh_A_EYI1BW401BR5QA | NBRI-0091 | 56 | NBRI_Gh_G001 | NBRI-0163 |
| 57 | NBRI_Gh_A_EYI1BW401BRUJS-1 | NBRI-0092 | 57 | NBRI_Gh_G018 | NBRI-0164 |
| 58 | NBRI_Gh_A_EYI1BW401CHO88 | NBRI-0093 | 58 | NBRI_Gh_G028 | NBRI-0165 |
| 59 | NBRI_Gh_A_EYI1BW401CHOJ0 | NBRI-0094 | 59 | NBRI_Gh_H007 | NBRI-0166 |
| 60 | NBRI_Gh_A_EYI1BW401CIU8I | NBRI-0095 | 60 | NBRI_Gh_H021 | NBRI-0167 |
| 61 | NBRI_Gh_A_EYI1BW401CKUXT | NBRI-0096 | 61 | NBRI_Gh_J007 | NBRI-0168 |
| 62 | NBRI_Gh_A2_191 | NBRI-0100 | 62 | NBRI_Gh_J015 | NBRI-0169 |
| 63 | NBRI_Gh_A2_462 | NBRI-0101 | 63 | NBRI_Gh_J025 | NBRI-0170 |
| 64 | NBRI_Gh_A2_5377 | NBRI-0102 | 64 | NBRI_Gh_J026 | NBRI-0171 |
| 65 | NBRI_Gh_B_2241 | NBRI-0103 | 65 | NBRI_Gh_K034 | NBRI-0172 |
| 66 | NBRI_Gh_B_EYI1BW404IEELX | NBRI-0104 | 66 | NBRI_Gh_L015(b) | NBRI-0173 |
| 67 | NBRI_Gh_B_EYI1BW404IQ9AX | NBRI-0105 | 67 | NBRI_Gh_L016 | NBRI-0174 |
| 68 | NBRI_Gh_B_EYI1BW404JGRFI | NBRI-0106 | 68 | NBRI_Gh_L025 | NBRI-0175 |
| 69 | NBRI_Gh_B2_2094 | NBRI-0109 | 69 | NBRI_Gh_L033 | NBRI-0176 |
| 70 | NBRI_Gh_B2_2245 | NBRI-0110 | 70 | NBRI_Gh_PA_11 | NBRI-0177 |
| 71 | NBRI_Gh_B2_2389 | NBRI-0111 | 71 | NBRI_Gh_PC_12 | NBRI-0178 |
| 72 | NBRI_Gh_C_1413 | NBRI-0112 | 72 | NBRI_Gh_PC_29 | NBRI-0179 |
| 73 | NBRI_Gh_C_2145 | NBRI-0113 | 73 | NBRI_Gh_PC_40-1 | NBRI-0180 |
| 74 | NBRI_Gh_C_7125 | NBRI-0114 | 74 | NBRI_Gh_PC_51-1 | NBRI-0181 |
| 75 | NBRI_Gh_C_EYT27PB01A00WQ | NBRI-0115 | 75 | NBRI_Gh_PC_54 | NBRI-0182 |
| 76 | NBRI_Gh_C_EYT27PB01A9DJZ | NBRI-0116 | 76 | NBRI_Gh_PC_56 | NBRI-0183 |
| 77 | NBRI_Gh_C_EYT27PB01A9GUT | NBRI-0117 | 77 | NBRI_Gh_PC_66 | NBRI-0184 |
| 78 | NBRI_Gh_C_EYT27PB01AOZ6G | NBRI-0118 | 78 | NBRI_Gh_PC_7(a) | NBRI-0185 |
| 79 | NBRI_Gh_C_EYT27PB01AZ4MQ | NBRI-0121 | 79 | NBRI_Gh_PC_7(b) | NBRI-0186 |
| 80 | NBRI_Gh_C_EYT27PB01B1E30 | NBRI-0122 | 80 | NBRI_Gh_PC_72-1 | NBRI-0187 |
| 81 | NBRI_Gh_C_EYT27PB01B3TPZ | NBRI-0123 | 81 | NBRI_Gh_PC_9(a) | NBRI-0188 |
| 82 | NBRI_Gh_C_EYT27PB01BK1E1 | NBRI-0124 | 82 | NBRI_Gh_PC_9(b) | NBRI-0189 |
| 83 | NBRI_Gh_C_EYT27PB01BT5N7 | NBRI-0125 | 83 | NBRI_Gh_PD_18 | NBRI-0190 |
| 84 | NBRI_Gh_C_EYT27PB01BYUKU | NBRI-0126 | 84 | NBRI_Gh_B004(a) | NBRI-0193 |
| 85 | NBRI_Gh_C_EYT27PB01CBAMC | NBRI-0127 | 85 | NBRI_Gh_B004 (b) | NBRI-0194 |
| 86 | NBRI_Gh_C2_2542 | NBRI-0130 | 86 | NBRI_Gh_C101 | NBRI-0196 |
| 87 | NBRI_Gh_D_EYT27PB02C1FO8 | NBRI-0131 | 87 | NBRI_Gh_D_EYT27PB02EESBJ(a) | NBRI-0203 |
| 88 | NBRI_Gh_D_EYT27PB02D2JWE | NBRI-0132 | 88 | NBRI_Gh_H040 | NBRI-0207 |
| 89 | NBRI_Gh_D_EYT27PB02D5TMM | NBRI-0133 | 89 | NBRI_Gh_PC_10 | NBRI-0208 |
| 90 | NBRI_Gh_D_EYT27PB02DBJ6P | NBRI-0134 | 90 | NBRI_Gh_B007(b) | NBRI-0210 |
| 91 | NBRI_Gh_D2_2099 | NBRI-0141 | 91 | NBRI_Gh_B008 | NBRI-0211 |
| 92 | NBRI_Gh_E_4745 | NBRI-0142 | 92 | NBRI_Gh_PC_82 | NBRI-0213 |
| 93 | NBRI_Gh_E_EYT27PB03F56SN | NBRI-0143 |  |  |  |
| 94 | NBRI_Gh_E_EYT27PB03FIO98 | NBRI-0144 |  |  |  |
| 95 | NBRI_Gh_E_EYT27PB03FOIX2 | NBRI-0145 |  |  |  |
| 96 | NBRI_Gh_E_EYT27PB03FTFEU | NBRI-0146 |  |  |  |
| 97 | NBRI_Gh_E_EYT27PB03FWL7V | NBRI-0147 |  |  |  |
| 98 | NBRI_Gh_E_EYT27PB03G94OB | NBRI-0148 |  |  |  |
| 99 | NBRI_Gh_E_EYT27PB03GGS4J | NBRI-0149 |  |  |  |
| 100 | NBRI_Gh_E_EYT27PB03GR05O | NBRI-0150 |  |  |  |
| 101 | NBRI_Gh_E_EYT27PB03GSS39 | NBRI-0151 |  |  |  |
| 102 | NBRI_Gh_E_EYT27PB03HBK3A-1 | NBRI-0152 |  |  |  |
| 103 | NBRI_Gh_E_EYT27PB03HBK3A-2 | NBRI-0153 |  |  |  |
| 104 | NBRI_Gh_E2_2382 | NBRI-0156 |  |  |  |
| 105 | NBRI_Gh_A_EYI1BW401CCYE8 | NBRI-0191 |  |  |  |
| 106 | NBRI_Gh_A2_5312 | NBRI-0192 |  |  |  |
| 107 | NBRI_Gh_B2_561-2 | NBRI-0195 |  |  |  |
| 108 | NBRI_Gh_C2_1595 | NBRI-0197 |  |  |  |
| 109 | NBRI_Gh_D_EYT27PB02C5AN1 | NBRI-0198 |  |  |  |
| 110 | NBRI_Gh_D_EYT27PB02DB09Y | NBRI-0199 |  |  |  |
| 111 | NBRI_Gh_D_EYT27PB02DHPDW | NBRI-0200 |  |  |  |
| 112 | NBRI_Gh_D_EYT27PB02DMO2Y-1 | NBRI-0201 |  |  |  |
| 113 | NBRI_Gh_D_EYT27PB02DMP7W | NBRI-0202 |  |  |  |
| 114 | NBRI_Gh_D2_733 | NBRI-0204 |  |  |  |
| 115 | NBRI_Gh_E_EYT27PB03FYW2W | NBRI-0205 |  |  |  |
| 116 | NBRI_Gh_E2_2159-1 | NBRI-0206 |  |  |  |
| 117 | NBRI_Gh_B_5458 | NBRI-0209 |  |  |  |
| 118 | NBRI_Gh_C_5476 | NBRI-0212 |  |  |  |
